# Biochemical and Structural Insights Concerning Triclosan Resistance in a Novel YX_7_K Type Enoyl-Acyl Carrier Protein Reductase from Soil Metagenome

**DOI:** 10.1038/s41598-019-51895-2

**Published:** 2019-10-28

**Authors:** Raees Khan, Amir Zeb, Kihyuck Choi, Gihwan Lee, Keun Woo Lee, Seon-Woo Lee

**Affiliations:** 1 Department of Applied Bioscience, Dong-A University, Busan, 49315 Republic of Korea; 2 Department of Biological Sciences, National University of Medical Sciences, Rawalpindi, 46000 Pakistan; 3 Division of Applied Life Science (BK21 Plus Program), Plant Molecular Biology and Biotechnology Research Center (PMBBRC), Research Institute of Natural Science (RINS), Gyeongsang National University, Jinju, 52828 Republic of Korea

**Keywords:** Environmental monitoring, Soil microbiology

## Abstract

Enoyl-acyl carrier protein reductase (ENR) catalyzes the last reduction step in the bacterial type II fatty acid biosynthesis cycle. ENRs include FabI, FabL, FabL2, FabK, and FabV. Previously, we reported a unique triclosan (TCL) resistant ENR homolog that was predominant in obligate intracellular pathogenic bacteria and Apicomplexa. Herein, we report the biochemical and structural basis of TCL resistance in this novel ENR. The purified protein revealed NADH-dependent ENR activity and shared similarity to prototypic FabI. Thus, this metagenome-derived ENR was designated FabI2. Unlike other prototypic bacterial ENRs with the YX_6_K type catalytic domain, FabI2 possessed a unique YX_7_K type catalytic domain. Computational modeling followed by site-directed mutagenesis revealed that mild resistance (20 µg/ml of minimum inhibitory concentration) of FabI2 to TCL was confined to the relatively less bulky side chain of A128. Substitution of A128 in FabI2 with bulky valine (V128) elevated TCL resistance. Phylogenetic analysis further suggested that the novel FabI2 and prototypical FabI evolved from a common short-chain dehydrogenase reductase family. To our best knowledge, FabI2 is the only known ENR shared by intracellular pathogenic prokaryotes, intracellular pathogenic lower eukaryotes, and a few higher eukaryotes. This suggests that the ENRs of prokaryotes and eukaryotes diverged from a common ancestral ENR of FabI2.

## Introduction

The last reduction step of the bacterial type II fatty acid synthesis (FASII) cycle is catalyzed by enoyl-acyl carrier protein reductase (ENR), which reduces enoyl-ACP to fully saturated acyl-ACP. Various coenzymes catalyzing this reaction include NADH, NADPH, and FMNH_2_ ^1^. Except for ENR, the majority of the other enzymes involved in the FASII cycle are relatively conserved among bacteria^2^. Various prototypical ENR isozymes of bacterial origin include FabI^3^, FabL^4^, FabV^5^, FabK^6^, and FabL2^7^. FabK is a flavin mononucleotide (FMN)-containing protein, whereas all other reported ENR isozymes belong to the short-chain dehydrogenase reductase (SDR) superfamily^8^. Despite sharing relatively low sequence identity (15–30%), the active site and specific sequence motifs for coenzyme binding are highly conserved in the various ENRs^9–11^.

The antimicrobial agent triclosan [5-chloro-2-(2,4-dichlorophenoxy)-phenol, TCL] prevents microbial growth by targeting ENR and thereby blocking FASII^12^. TCL has been being used in a variety of consumer and personal care products for years^13–15^ with the supposition that TCL-containing products provide protection against the majority of pathogenic bacteria. However, various intrinsic or acquired TCL resistance mechanisms have been reported in bacteria. These include the elevated expression of ENR^16^, presence of TCL tolerant and/or mutated ENR^17^, cell membrane modification^18^, various efflux pumps^16,19^, enzymatic degradation of TCL^20^, novel and unique ENRs^21^, and unknown TCL resistance determinants^21^. Furthermore, TCL selective pressure has been associated with induced co- or cross-resistance to other antimicrobials^17,21–26^. More recently, various environmental and public health concerns have been linked to the excessive use of TCL^13,14^.

FabI is the only known ENR that is sensitive to TCL. However, FabI is prone to mutations that are due to substitutions. While TCL acts as an inhibitor of *Escherichia coli* carrying FabI at the picomolar level (*K*_*i*_ = 38 pM) by mimicking its original substrate^27^, substitutions at various key residues of the enzyme can result in significant TCL resistance^21,28–30^. The level of TCL resistance varies in *E. coli* and *Staphylococcus aureus* dependent on the key residues^28–30^. ENRs that include FabV^5^, FabL2, and FabG-like ENR homologs^21^ are completely resistant to TCL. The FabL ENR confers partial resistance to TCL. Conversely, the FabK ENR confers moderate^21^ to complete TCL resistance^6^. Since ENRs are crucial for bacterial survival and growth, they have been potential antimicrobial targets for many years, and a variety of synthetic ENR inhibitors have been marketed, are in development, or are being examined in trials^31,32^.

Previously, we reported a mild TCL-resistant ENR homolog (KT860426.1; AOO54553.1) obtained from the soil metagenome. This ENR had a different YX_7_K type catalytic domain (where X_7_ are any seven amino acids between tyrosine (Y) and Lysine(K)) and was capable of complementing ENR activity in the temperature sensitive *E. coli* mutant JP1111 (*fabI* ^ts^)^21^. Since this ENR was similar to the prototypic FabI, we designated this metagenome-derived YX_7_K type ENR as FabI2. The FabI2 ENR is unique because it is highly similar to FabI2 homologs and predominates in obligate intracellular pathogenic bacteria and the Apicomplexa phylum of eukaryotic obligate intracellular pathogens^21^.

In this study, we analyzed the enzyme activity and structure of the FabI2 ENR, and explored the basis of TCL resistance. Phylogenetic analysis, biochemical characterization, and molecular simulation studies support the idea that the novel FabI2 ENR divergently evolved from a short-chain dehydrogenase reductase family. A unique catalytic active site of FabI2 has been highly conserved and shared by prokaryotes and eukaryotes. Since this FabI2 ENR is prevalent in obligate intracellular pathogenic bacteria and Apicomplexa, the ENR is an important focus for healthcare and drug discovery studies.

## Materials and Methods

### Bacterial strains, plasmids, culture condition, and general DNA manipulation

*Escherichia coli* strains DH5α, EPI300, and BL21 (DE3) were cultured at 37 °C in broth or solid Luria-Bertani (LB) medium amended with the desired antibiotics. The antibiotics included TCL, 0–600 µg/ml; chloramphenicol, 50 µg/ml; ampicillin, 100 µg/ml; and kanamycin, 50 µg/ml. TCL was commercially obtained from Sigma-Aldrich Co. (St. Louis, MO, USA). Most of the recombinant DNA manipulations were carried out as previously described^33^. Oligonucleotide synthesis and DNA sequencing were performed commercially at the MacroGen sequencing facility (Seoul, Korea). Comparative analysis of the nucleotide and amino acids sequences was carried out using the publicly available BLAST and ORF finder online services at the National Center for Biotechnology Information (NCBI http://blast.ncbi.nlm.nih.gov). Multiple alignment analysis was performed using BioEdit and GeneDoc software.

### Phylogenetic analysis

Phylogenetic analysis for the metagenomic FabI2 ENR was carried out as described previously^21^ using amino acid sequences of FabI2 ENR and its homologs. These included previously known, well-characterized prototypical FabL, FabL2, FabI, FabV, and FabK ENRs and their homologs retrieved from the UniRef50 database. The search of this database resulted in sequence homologs that were at least 50% identical to the cluster sequence of the database, and the top 10 scoring entries for individual homology search were selected. The identified sequences were compiled along with the closely related corresponding prototypic ENRs and metagenomic FabI2, and redundant sequences were removed using the online Decrease Redundancy program^34^. MEGA 6 was used for sequence alignment and phylogenetic tree construction^35^ using the MUSCLE algorithm^36^. The alignment output was analyzed in MEGA 6 by utilizing the maximum likelihood method in combination with nearest-neighbor-interchange strategy, in which gaps present in less than 50% of the sequences were deleted. The confidence of the method was evaluated using 500 bootstrap replicates. Additionally, a phylogenetic tree based on the fast minimum evolution method was constructed against the 250 closest hits (sharing 66–84% identity with FabI2) of NCBI blastp results using the “Distance tree of results” option.

### Expression and purification of FabI2 ENR

The *fabI2* gene from the pAF1 clone^21^ was amplified using the gene-specific forward primer (5′-TAGTGAGGT**GGATCC**TATGGTTTCAATGAATCTCAAAGG-3′) and reverse primer (5′-TTTACCGTCATGTTCGATCGCGCA**GTCGAC**GTTGGCGA-3′) containing *Bam*HI and *Sal*I restriction sites (which are in bold, underlined), respectively. Restriction digestion of the PCR products was performed with appropriate restriction enzyme followed by ligation into the corresponding restriction enzyme site in the expression vector pET-30b(+) to construct a recombinant vector pAF1–5. The pAF1–5 vector was subsequently introduced into competent *E. coli* BL21 (DE3), which were grown to an optical density at 600 nm (OD_600_) of 0.5 at 37 °C in 200 ml LB broth containing kanamycin. Protein expression was induced by adding isopropyl β-D-1-thiogalactopyranoside (IPTG, 1 mM) at the late exponential phase. *E. coli* with over-expressed fusion proteins were harvested and processed for protein purification by first re-suspending the bacteria in 5 ml binding buffer (20 mM Tris-Cl; 0.5 M NaCl; 40 mM imidazole; pH 8.0). The bacteria were sonicated (Sonic Dismembrator Model 500; Thermo Fisher Scientific, Waltham, MA, USA) for 2 min using 5-sec pulses with intervening 10-sec intervals. Centrifugation was performed at 3,500 × g and 6 min at 25 °C. The supernatant was collected and re-centrifuged at 17,000 × g for 10 min at 25 °C. The resulting supernatant was finally filtered through a 0.45 µm pore size membrane filter. An AKTA prime liquid chromatography system (GE Healthcare, Buckinghamshire, UK) supplied with the His Trap™ HP affinity column (1 ml bed volume; GE Healthcare) was used to purify the fusion protein. The purified fusion protein of the expected size was finally confirmed using sodium dodecyl sulfate-polyacrylamide (SDS-PAGE).

### Analysis of enzyme activity

The deduced amino acid sequences of the metagenomic FabI2 protein are very similar to prototypic FabI from *E. coli* and FabI ENRs from obligate intracellular pathogenic bacteria and Apicomplexa, and *fabI2* complements ENR activity in a *fabI* mutant of *E. coli*^21^. To test for ENR activity for the FabI2 protein and to determine the optimum reaction conditions and Michaelis–Menten kinetics, we performed enzymatic assays with the purified fusion protein. All ENR activity assays were performed similarly as previously described^37^ with a few modifications. Briefly, ENR activity was determined in a 100 µl reaction mixture containing crotonyl-CoA (200 µM), NADH/NADPH (250 µM), purified protein (450 nM), and 100 mM sodium phosphate buffer (pH 7.0) at 25 °C. The progress of the enzymatic reactions was monitored using a DU®730 Life Science UV/Vis spectrophotometer (Beckman Coulter Inc., Fullerton, CA, USA) by monitoring at 340 nm every 30 sec for 3 min. The substrate crotonyl-CoA and cofactors NADH and NADPH were obtained from Sigma-Aldrich. The protein showed ENR activity only with NADH; therefore, NADH was used for all subsequent enzyme assays. The K_m_ value for the purified FabI2 protein with crotonyl-CoA substrate was determined. For this, a 100 µl reaction mixture containing 100 nM of purified protein was supplemented with 200 μM of cofactor NADH and varying concentrations of substrate crotonyl-CoA (3, 6, 12, 24, 36, or 48 μM). The K_m_ value for the cofactor NADH was similarly determined; the reaction mixture contained 100 nM of the FabI2 protein, 60 μM of substrate crotonyl-CoA and various concentrations of cofactor NADH (5, 10, 15, 20, 30, 50, and 75 μM). This reaction mixture was incubated for 10 min at 25 °C. The linear phase of the reaction curve was used to calculate the initial reaction velocity, and the K_m_ values both for substrate and cofactor were calculated by fitting the data into the standard Michaelis–Menten equation. All kinetic reactions were performed in triplicate, and the reaction progress was measured at 340 nm. To select the optimal buffer and pH for the protein activity, the enzyme reaction was carried out using various 100 mM sodium citrate buffer at pH 3.2, 4.2, 5.2, and 6.2, and 100 mM sodium phosphate buffer at pH 6.5, 7, 7.5, and 8.0.

### Site-directed mutagenesis

Although the FabI2 ENR contained a unique YX_7_K type catalytic domain, it shared relatively high homology with the prototypical FabI (30%) and FabL (27%) ENRs. Therefore, we assumed that the FabI2 ENR might confer resistance to TCL using a similar mechanism as FabI ENR, namely point mutations with amino acid substitutions at key enzyme residues that include G93V, G93A, G93S, M159T, F203L, F203C, F203A, and F203V (amino acid numbering was according to *E. coli* FabI ENR) could lead to various levels of TCL resistance. Among those, substitution at the G93 position was the most significant substitution that confers the highest TCL resistance^21^. To test whether an amino acid substitution at the key enzyme site of G93V could confer TCL resistance, site-directed mutagenesis was performed using overlap extension PCR (Supplementary Fig. S1). Two PCR procedures were performed to amplify the overlapping fragments A and B of the FabI2 encoding gene containing the desired point mutation from pAF1–5. The following primer pairs were used for these PCR reactions; FWYX7-A/RVYX7-A (FWYX7-A: 5X7GCCCCTAA**GGATCC**CATAAGCCTAAACCCT-3T and RVYX7-A: 5X7**GACAAGGGAGTGAACCAGG**ATATCGATT-3ATCGATTFWYX7-B/RVYX7-B (FWYX7-B: 5X7**CCTGGTTCACTCCCTTGTC**AATGGTCCTGA-3T and RVYX7-B: 5X7TGCACTCGACGTTGGCGATAAATA**GGATCC**AC-3′), respectively. The nucleotides sequences indicated in bold are complementary to each other, whereas those in bold and underlined are the *Bam*HI restriction sites. The PCR conditions comprised initial denaturation at 95 °C for 3 min; 30 cycles of denaturation at 95 °C for 1 min, annealing at 63 °C (for amplifying fragment A and B) for 30 s, and extension at 72 °C for 30 s; and a final extension at 72 °C for 5 min. The amplified PCR products A and B were gel-purified. To fuse fragments A and B, a second PCR was performed where both fragments were used as templates in equimolar concentrations without adding any primers with an initial denaturation at 95 °C for 5 min; 10 cycles of denaturation at 95 °C for 1 min, annealing at 63 °C for 30 s, and extension at 72 °C for 1 min; and a final extension at 72 °C for 5 min. The fusion product was subsequently amplified by adding primers FWYX7-A and RVYX7-B to the reaction mixtures (Supplementary Fig. S1) using an initial denaturation at 95 °C for 3 min; 20 cycles of denaturation at 95 °C for 1 min, annealing at 63 °C for 30 s, and extension at 72 °C for 1 min; and a final extension at 72 °C for 5 min. The purified fusion product was treated with *Bam*HI and cloned into the pUC119 vector, followed by transformation into *E. coli* DH5α. The A128V substitution of the FabI2 was further confirmed by DNA sequencing and tested for TCL resistance as previously described^21^. The mutated version of FabI2 was designated as mFabI2.

### Complementation

To confirm whether mFabI2 retained ENR activity, a complementation experiment was performed. The recombinant pUC119 plasmid carrying *mfabI2* was transformed into *E. coli* mutant JP1111, which has a mutation in *fabI* that renders it unable to grow at the high temperature of 42 °C^38^. *E. coli* JP1111 carrying *mfabI2* were allowed to grow in triplicate on LB agar amended with ampicillin (100 µg/ml) at 30 °C and 42 °C for 48 h. Growth at 42 °C suggested complementation of FabI ENR activity by the *mfabI2*.

### TCL resistance test

The growth and TCL resistance assays of *E. coli* DH5α carrying either *fabI2* or *mfabI2* were performed in LB broth and LB agar supplemented with ampicillin (100 µg/ml) and varying concentrations of TCL (0–600 µg/mlL). The positive control was *E. coli* DH5α expressing the prototypical FabI ENR of *E. coli* K-12 (NP_415804.1) in pUC119. The negative control was *E. coli* DH5α carrying empty pUC119 vector. Bacterial growth was assessed using the aforementioned DU730 Life Science UV/Vis spectrophotometer by measuring OD_600_ for 96 h.

### Homology modeling of FabI2 and mFabI2

Homology modeling was used to create an atomic model of the target protein from crystal structures of evolutionarily related proteins^39^. To identify a suitable template, the amino acid sequence of FabI2 was aligned against all the available structures of PDB using blastp and the position-specific iterative basic local alignment search tool (PSI-BLAST) in NCBI. The selected template was used to construct a FabI2 model using the MODELLER program that was implanted in Discovery Studio *v*4.5 (BIOVIA Corp., San Diego, CA, USA). The quality of FabI2 model was assessed by the Discrete Optimized Protein Energy (DOPE) score as previously described^7^.

Since the homology model does not reflect the physiological conformation and/or orientation of the residues of the target protein, the FabI2 model was subjected to molecular dynamics (MD) simulation. The simulation was performed using the GROMACS *v*5.0.7 package (www.gromacs.org/Downloads)^40^ with CHARMm36ff (mackerel.umaryland.edu/charm_ff.shtml)^41,42^ as previously described^7^. Briefly, the system was solvated in an octahedral box with the transferable intermolecular potential with 3 points (TIP3P) water model^43^. Sodium ions (Na^+^) were added to neutralize the system. Before simulation, energy minimization (with maximum force of 10 kJ/mol/nm) was carried out using the steepest descent algorithm to avoid steric clash. The Particle Mesh Ewald approach was employed for long-range electrostatics^44^ with a cutoff distance of 10 Å. The system was equilibrated in two phases. First, the system was simulated for 500 ps under a constant number of particles (NVT) at 300 K. The temperature was maintained using a V-rescale thermostat^45^. Second, the system was simulated under constant pressure (NPT) for 500 ps using the Parrinello-Rahman barostat to equilibrate at an isotropic pressure of 1.0 bar^46^. The equilibrated system was subjected to a 10-ns production run. During data collection, the V-rescale thermostat and Parrinello-Rahman barostat were used to maintain the temperature and pressure at 300 K and 1.0 bar, respectively.

The representative structure of FabI2 was extracted from last 6-ns trajectory. The stereochemical quality and accuracy of the MD refined FabI2 model were confirmed by validation servers, including ProSAweb (https://prosa.services.came.sbg.ac.at/prosa.php) and the SAVES server (http://services.mbi.ucla.edu/PROCHECK/). The MD refined model of FabI2 was used to generate mFabI2 by the Build Mutants module of Discovery Studio *v*4.5. The best model was selected by DOPE score. The mutant model was refined by MD simulation and validated using the same protocol as described for FabI2.

### MD simulation of TCL into FabI2 and mFabI2

To investigate the molecular interaction pattern of TCL with FabI2 and mFabI2, molecular docking was carried out using the Genetic Optimization of Ligand Docking *v*5.2.2 program^47^. The validated structure of target (FabI2 and/or mFabI2) was prepared as the protein structure for the docking simulation. The binding site of the target was traced from its conserved residues (A128, Y188, M192, and K196) and TCL coordinates using the Define and Edit Binding Site protocol, implanted in Discovery Studio v4.5. The two-dimensional (2D) structure of TCL was drawn in Accelrys *v*4.2, converted to a three-dimensional (3D) structure by Discovery Studio v4.5, and prepared as a mol_2_ file for docking simulation. The docking results were analyzed using the ChemPLP (Piecewise linear potential) score as a scoring function^48^. The best-docked pose was selected by high dock score, conformational stability of TCL, and hydrogen bond interactions with catalytic residue(s).

### Accession number(s)

The nucleotide accession number for the nucleotide sequences of metagenomic pAF1–5 clone, carrying the *FabI2* gene has been deposited in the GenBank database under accession number KT860426. The protein ID for FabI2 ENR is AOO54553.1.

## Results and Discussion

### Phylogenetic analysis of FabI2 and prototypic ENRs

ENR catalyzes the crucial final reduction step of the bacterial FASII cycle and is thus indispensable for bacterial growth and survival^49^. Structural similarity comparisons revealed that the deduced amino acid sequences of metagenomic FabI2 protein shared significant identity (46–77%) with ENR homologs of obligate intracellular pathogenic bacteria and the phylum Apicomplexa obligate intracellular pathogens, whereas it shared lower identity with prototypical FabI (30%), FabL (27%), and FabL2 (24%) ENRs, and did not share any significant identity with other well-known prototypical ENRs (Supplementary Table S1). Moreover, the FabI2 protein complemented the ENR activity in a conditional mutant of *E. coli* JPP1111 carrying the *fabI ^ts^ mutation. FabI2 also conferred moderate levels of TCL resistance (20 µg/ml), when expressed in an alternative host *E. coli*^21^.

**Figure 1:**
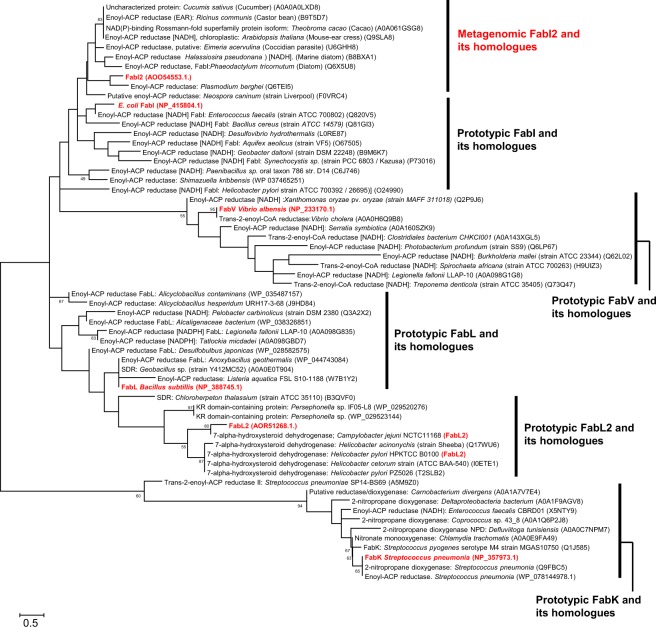
Metagenomic FabI2 ENR and its homologs cluster as a separate clade from other closely related prototypic ENRs. Phylogenetic tree of FabI2 with prototypic FabL2, FabL, FabV, FabI, and FabK (shown in bold red) ENRs. Maximum likelihood analysis was performed with well-characterized FabL2, FabL, FabV, FabI, and FabK (in bold) and their homologs with >50% sequence identity from the Uniref50 database. Bootstrap values are shown for each node that had >50% support in a bootstrap analysis of 500 replicates. The scale bar represents 0.5 estimated amino acid substitutions per residue.

Phylogenetic analysis of the FabI2 protein with all other prototypic ENRs and closely related FabI proteins and their homologs revealed that the FabI2 type ENRs clustered as a separate clade (Fig. 1). This suggested that the FabI2 type metagenomic ENR may be evolutionarily close to the FabI type ENRs, and may have potentially diverged from either closely related FabI and FabV ENRs, or FabL ENRs. In addition, the distance tree of the closest top 250 hits (sharing 66–84% identity to FabI2) revealed that this ENR was shared by eukaryotes and prokaryotes (Supplementary Fig. S2). To our knowledge, no bacterial ENR reported to date is shared by both prokaryotes and eukaryotes.

### FabI2 protein displays NADH-dependent ENR activity

**Figure 2:**
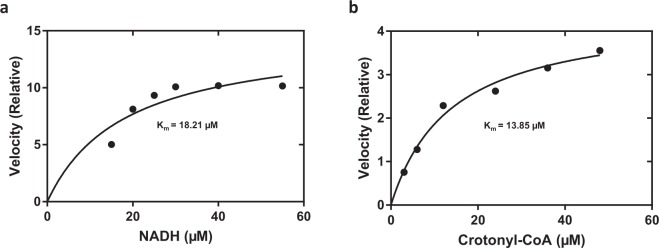
Biochemical analysis of the metagenomic FabI2 ENR. Initial velocities of the reaction were determined in duplicate as a function of varying concentrations of (**a**) the cofactor (NADH) and (**b**) substrate crotonyl-CoA. Data were fit to the Michaelis–Menten equation choosing the nonlinear regression equation using GraphPad Prism version 7. The fitted line and K_m_ values are shown.

SDS-PAGE analysis confirmed the expected overexpression of FabI2 fusion protein and purification of the protein with the expected size (37.74 kDa) (Supplementary Fig. S3). To assess the ENR enzyme activity of the purified protein, oxidation of the NADH/NADPH cofactors to NAD/NADP was monitored at 340 nm. Maximum protein activity was observed in 100 mM sodium phosphate buffer (pH 7.0) (Supplementary Fig. S3c); thus, it was used for further kinetic assays of the protein. Relatively lower enzyme activity was observed in 100 mM sodium citrate buffer (pH 3.2–6.2) (Supplementary Fig. S3). The purified protein catalyzed the turnover of NADH (K_m_ 18.21 µM) (Fig. 2a) to NAD in the presence of substrate crotonyl-CoA (K_m_ = 13.85 µM) as a substrate (Fig. 2b). Nearly the same K_m_ values have been reported for metagenomic FabL2 of *Chlamydia trachomatis* and *E. coli* ENRs^4,7,37^. However, the K_m_ for FabI2 was relatively higher than those reported for some ENRs^27,50^. The purified FabI2 protein utilized only NADH as a cofactor and did not show any activity with NADPH (data not shown).

Sequence comparisons of the metagenomic FabI2 to other well-known prototypic ENRs revealed relatively higher identity to the prototypic FabI from *E. coli* (30%) and with *C. trachomatis* ENR (67%). Both use NADH as a cofactor^3,37,51^. FabK is another example of an ENR that uses NADH as a cofactor^52^. However, FabK is not an SDR and belongs to a completely different class of FMN dependent proteins that do not share any sequence similarity to FabI2. The *kcat* values (Supplementary Table S2) for FabI2 with the substrate crotonyl-CoA (0.19396 µM/min) and NADH (0.9546 µM/min) were similar to those reported previously^7,53^ but were lower than those of ENR from *E. coli* and *C. trachomatis*^37,54^. Taken together, these biochemical analyses confirmed that the metagenomic FabI2 is an NADH-dependent ENR. The metagenomic FabI2 ENR shares some structural similarity with the prototypic FabI and FabL ENR, but it possesses a unique YX_7_K type catalytic domain.

### Structure prediction and validation of FabI2

**Figure 3:**
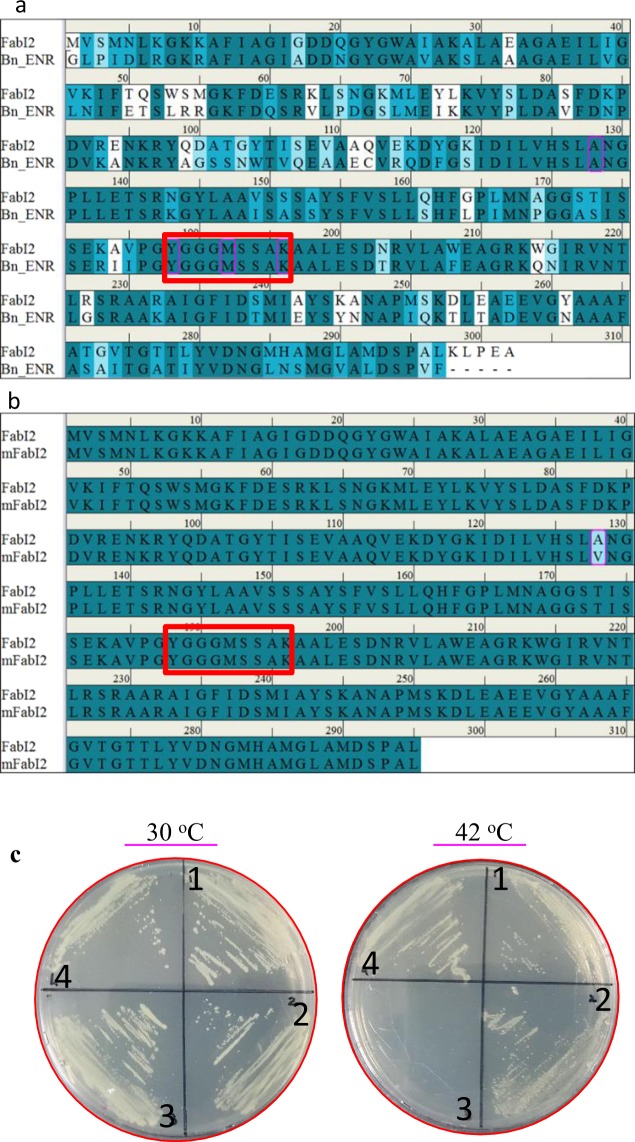
Sequence alignment of FabI2 with template (BnENR) and mFabI2. (**a**) Sequence alignment of FabI2 and BnENR (PDB ID: 1D7O). The crystallographic structure was resolved as an enoyl-acyl carrier protein reductase of *Brassica napus*. The YX_7_K catalytic active site is shown as a rectangular red box, whereas the catalytic and substrate binding residues are shown as magenta boxes. (**b**) Sequence alignment of FabI2 and mFabI2. The mutated residue (A128V) is shown as a rectangular box. (**c**) Complementation analysis of mFabI2 ENR. Each plate was divided into four separate sections. Section 1 is JP1111 with FabI2 in pUC119, section 2 is JP1111 with mFabI2 in pUC119, section 3 is JP1111 with pUC119 only, and section 4 is JP1111 carrying *E. coli* FabI in the pUC119 vector. Plates were incubated at 30 °C or 42 °C for 48 h.

The blastp search of deduced amino acids sequence of FabI2 showed that FabI2 share high sequence identity to the ENR of *Brassica napus* (BnENR) (67%) (Fig. 3a) of which the crystal structure has been resolved in complex with TCL (PDB ID:1D7O). FabI2 likely possesses potentially similar TCL binding residues (Fig. 3b), and it complemented ENR activity (Fig. 3c). The co-crystal structure of plant BnENR bound NADH and TCL has been resolved to a resolution of 1.9 Å^55^. Sequence comparison between BnENR and FabI2 suggested that the catalytic and substrate binding residues of the plant BnENR (A211, Y271, M275, and K279) are well conserved in FabI2 (A128, Y188, M192, and K196) (Fig. 3a). This suggested that the plant BnENR might be a suitable template for homology modeling of FabI2. For FabI2, a total of ten models were generated, and the top-ranked model with lowest DOPE score (−30592.28) was selected.

**Figure 4:**
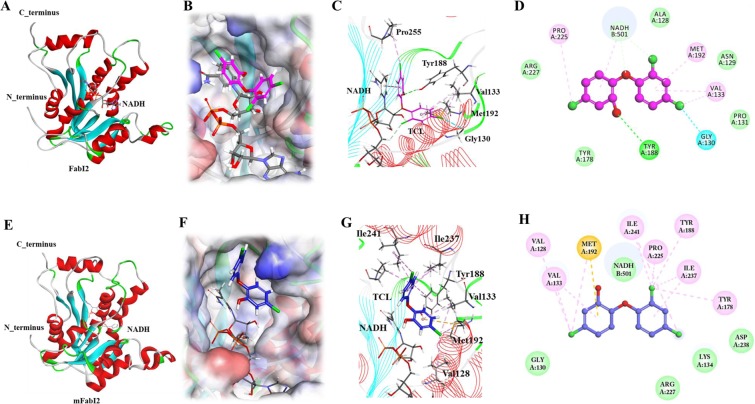
Homology model and docking of FabI2, mFabI2 and TCL. (**A**) Optimized model of FabI2. Alpha-helices flank the parallel β-strands and form an ENR-like architecture. NADH is shown as a stick model. (**B**) Three-dimensional (3D) view of docked TCL in FabI2. TCL occupies and orients the inhibitory mode in the active site of FabI2. (**C**) 3D view of molecular interactions of the FabI2-TCL complex. TCL is depicted as magenta stick representation. The phenolic ring of TCL and nicotinamide ring of NADH are stacked by face-to-face weak hydrophobic interactions. The phenolic oxygen of TCL forms an H-bond (green dash) with Y188 of FabI2. (**D**) Two-dimensional (2D) representation of molecular interactions between FabI2 and TCL. (**E**) MD optimized model of mFabI2. NADH is shown as stick representation. (**F**) 3D view of docked TCL in mFabI2. TCL cannot occupy and orient in an inhibitory mode in the active site of mFabI2. TCL is shown as blue stick representation. (**G**) 3D view of molecular interactions of the mFabI2-TCL complex. The orientation and binding pattern of TCL are completely inverted, where the di-chloro ring of TCL occupies the wide and shallow region of the catalytic cavity of mFabI2. TCL cannot form an H-bond with catalytic residues of mFabI2. TCL is shown as blue stick representation. (**H**) 2D representation of molecular interactions between mFabI2 and TCL.

The FabI2 model in complex with NADH was refined by a 10-ns MD simulation. Structural elucidation of the refined model revealed that FabI2 has 7-stranded parallel β-sheets (β1 to β7) flanked on both sides by six α-helices, with three on each side, to form an NADH-binding Rossman-like fold^55,56^ (Fig. 4A). Moreover, the interior and posterior corners of the aligned β-sheets were sealed by α-helices (Fig. 4A). Such a topological arrangement of β-sheets and α-helices has been previously described for other ENRs structures^30,55–59^. Our results suggested that NADH is aligned with FabI2 close to the active site residues to produce a well-conserved cavity for the binding of substrate or inhibitor (Fig. 4A). TCL binds to active site of ENRs in association with NADH^57,60^.

Validation of the stereochemical quality of FabI2 revealed that 90% of the FabI2 residues occupied the most favored region of the Ramachandran Plot (Supplementary Fig. S4a). The distribution of the remaining residues was also observed within the acceptable range. These observations suggested that the phi (Φ) and psi (ψ) backbone dihedral angles are reasonably accurate^61^. Furthermore, the Z-score (−6.84) of FabI2 was within the range of Z-score of experimentally determined structures, suggesting its native folding (Supplementary Fig. S4b)^62^.

### TCL inhibits FabI2

Docking of TCL with FabI2 displayed the highest docking score of 54.73. Our computational analyses suggested that TCL could occupy the active site of FabI2 and form stable interactions with active catalytic residues (Fig. 4B,C). This binding orientation pattern of TCL is well conserved in the active site of ENRs^30,55,56^. Furthermore, our docking results suggested that the phenolic hydroxyl group of TCL formed a hydrogen bond with the phenolic oxygen of Y188 (Fig. 4C,D). The ENR inhibition by antagonists via the targeting of tyrosine residue at this particular position has been reported^12,29,37,55–57^. In contrast, the present docking results suggest that TCL could not establish a hydrogen bond (H-bond) with NADH (Fig. 4C,D). In contrast, other reports described the formation of H-bonds between TCL and NADH^12,29,30^. We speculate that A128 in FabI2 is most likely responsible for weakening TCL binding by the absence of H-bond formation with NADH. Our results echo those of Levy *et al*.^56^, who suggested that replacement of G93A of FabI in *E. coli* K12 (the corresponding native residue is A128 in FabI2) leads to remarkable weakening of TCL binding. Docking of TCL to BnENR generated a similar result, showing that TCL formed an H-bond with Y271 because BnENR contain A211 at the corresponding position of G93 in *E. coli* K12 ENR^55^. Furthermore, our docking results indicated that the phenol ring of TCL formed a face-to-face interaction with the nicotinamide ring of NADH, allowing weak phi-phi stacking interaction (Fig. 4C). In contrast, the FabI ENR of *E. coli* K12 displays extensive stacking interactions between the phenol ring of TCL and the nicotinamide ring of NADH^56^. Furthermore, Stewart *et al*.^30^ reported that interactions between EnvM, an ENR of *E. coli*, and TCL pre-dominantly represent a hydrophobic-hydrophobic interaction. The only exception is the formation of a strong hydrogen bond between the Y156 and the hydroxyl group of TCL. Therefore, we speculate that poor stacking of NADH and TCL is the cause of the topological effects of A128 of FabI2, which renders FabI2 partially resistant to TCL. Overall, our computational analyses suggested that TCL binds and inhibits FabI2 through the same mechanism to inhibit other members of ENR family. The lack of H-bond and weak π-π stacking interactions of FabI2 with NADH may also explain the partial inhibition of ENR activity by TCL.

### Structure model generation of mFabI2 and validation

A total of ten mFabI2 models were generated from the FabI2 structure by mutating the alanine at position 128 (Ala128) to a valine residue in the Discovery Studio v4.5 software. The best model of mFabI2 had the lowest DOPE score (−27821.36). This model was used for the 10-ns MD simulation. The MD refined model of mFabI2 revealed a similar structure as explained earlier for FabI2 (Fig. 4E). Validation of mFabI2 suggested that approximately 92% of the residues occupied the most favored region in the Ramachandran plot (Supplementary Fig. S4c). The distribution of other residues also occupied the acceptable regions of the Ramachandran plot. Such a pattern of residue distribution suggests that the Φ and ψ backbone dihedral angles of mFabI2 are reasonably accurate^61^. Furthermore, the Z-score of mFabI2 was in the range of experimentally determined PDB structures (Supplementary Fig. S4d)^62^. The collective results suggested that the target mutation did not denature the structure of mFabI2 and that the native folding of the protein was preserved.

### TCL does not inhibit mFabI2

The TCL binding site of mFabI2 was determined by its superimposition on the FabI2 model. The molecular docking of TCL in the active site of mFabI2 produced a remarkably low docking score of 42.62. Furthermore, the binding conformation of TCL in the active site of mFabI2 was altered compared to its inhibitory conformation in the wild-type FabI2 (Fig. 4F). The docking results demonstrated that the bulkier di-chloro-phenol group of TCL occupied the interior position of the broad and shallow cavity of mFabI2 (Fig. 4F). We speculate that this altered binding conformation of TCL might be caused by its flipping due to the bulkier side chain of valine residue (V128) of mFabI2. Our results substantially agree with the description by Levy *et al*.^56^ that valine substitution at position G93 in ENR of *E. coli* resulted in pronounced resistance to TCL. Moreover, the molecular interactions of TCL with mFabI2 were drastically altered from its interaction with FabI2. TCL abolished the H-bond formation with Y188 in mFabI2 (Fig. 4G,H). Furthermore, other hydrophobic and van der Waals interactions were not well established between the TCL and mFabI2 (Fig. 4F). These collective results suggested that the inhibition of ENR activity by TCL requires a particular conformation in the active site of target ENR. Consequently, specific mutation(s) in ENRs can disturb or completely abolish the binding affinity of TCL to the mFabI2, which would possibly alter the enzyme inhibition by TCL.

### Comparison of TCL binding between BnENR, FabI2, and mFabI2

Since BnENR (PDB ID:1D7O) (Supplementary Fig. S5a) was used as a template for homology modeling of FabI2, the bonding pattern of TCL was compared in the active site of BnENR, FabI2, and mFabI2. Superimposition of BnENR and FabI2 in complex with TCL suggested that TCL bound FabI2 by the same mechanism as in BnENR (Supplementary Fig. S5b). Our docking results also suggested that TCL occupied the active site of FabI2. TCL obtained the same binding conformation in the active site of FabI2 as it binds BnENR, and inhibited FabI2. Conversely, the superimposition of BnENR and mFabI2 demonstrated that, despite occupying the active site of mFabI2, TCL could not inhibit mFabI2 due to the loss of preserved binding pattern (hydrogen bond formation with Y188 of mFabI2 and hydrophobic interaction with NADH) (Figs 4H and S5c). Our observations suggested that TCL requires a compatible conformation and the establishment of conserved molecular interactions to inhibit ENR(s). Furthermore, the superimposition of FabI2 and mFabI2 suggested that the binding conformation of TCL allowed the inhibition of FabI2 only (Supplementary Fig. S5d).

### FabI2 type ENR homologs are strictly conserved and shared among obligate intracellular pathogenic bacteria, apicomplexa, and higher eukaryotes

Multiple alignment analyses revealed that the metagenomic FabI2 has a unique YX_7_K type catalytic domain (Fig. 3 and Supplementary Fig. S6). This domain was previously reported to be specific to higher eukaryotes, including plant origin FabI ENR^1^. However, to the best of our knowledge, no TCL resistance has been reported for YX_7_K type ENRs. We previously reported that the FabI2 type ENR is strictly conserved among obligate intracellular pathogenic bacteria and Apicomplexa^21^. We speculated that the closely related homologs of FabI2 from those organisms share a similar YX_7_K type catalytic domain. Comparative analysis based on multiple alignments revealed the presence of the similar YX_7_K type catalytic domain (Supplementary Figs S7 and S8) in a variety of closely related homologs of FabI2. The strict amino acid conservation of this YX_7_K type catalytic domain (Supplementary Fig. S7) was apparent among obligate intracellular pathogenic bacteria, obligate intracellular pathogenic lower eukaryotes (Apicomplexa), and higher eukaryotes of plant origin (Supplementary Data 1). This suggests a prokaryotic origin of FabI2, which might have evolved to higher eukaryotic ENRs.

### FabI2 share a similar mechanism of TCL resistance to prototypic FabI

Amino acid sequence comparisons and phylogenetic and bioinformatics analyses revealed a high identify between the metagenomic FabI2 and the prototypical FabI ENR (30%, Supplementary Table S1), while FabI2 harbored a unique YX_7_K type catalytic domain (Supplementary Table S3). Furthermore, examination of the docking of TCL into the active site of mFabI2 revealed that TCL could not adopt an inhibitory conformation or H-bond with Y188 of the mFabI2. Therefore, we speculated that FabI2 ENR might share a similar TCL resistance mechanism as reported previously for prototypic FabI ENR, where the G93V substitution of FabI (corresponding to A128 of FabI2) was shown to confer multifold resistance to TCL^21^. To test whether the A128V substitution could confer similar resistance to TCL, site-directed mutagenesis was performed to replace the A128 of FabI2 with valine (Supplementary Fig. S1). As expected, the A128V substitution resulted in increased resistance to TCL in mFabI2 (600 µg/ml; maximum level tested) as compared to the wild-type FabI2 (minimum inhibitory concentration, 20 µg/ml) (Supplementary Fig. S9 and Supplementary Table S4). Regardless of the point mutation in mFabI2, the complementation test revealed that the mFabI2 maintained its original ENR activity (Supplementary Fig. S4c). These results indicated that mFabI2 likely confers TCL resistance in a similar manner as the prototypical FabI, and that both possess similar TCL binding pockets. Moreover, the substitution of simple amino acids (alanine, for instance) with bulky side chains amino acids, such as G93V, lead to steric interference in TCL binding, and ultimately results in significant resistance to TCL^21^.

ENRs crucial for bacterial survival and growth. Thus, the enzymes have been targeted as potential antimicrobial targets against pathogenic microorganisms. Various TCL-based or non-TCL-based ENR inhibitors are commercially available, under developed, or being evaluated in trials^31^. Considering the recent reports and interest for developing TCL-based antimalarial inhibitors^63^ capable of targeting both *Plasmodium falciparum* ENR (It is homologous (49% identical) to FabI2 and harbors the YX_7_K type domain.) and dihydrofolate reductase, resistance associated with amino acid substitutions of these YX_7_K type ENRs need to be considered prior to developing TCL-based antimalarial drugs.

## Conclusions

The results indicate that FabI2 is a mildly TCL tolerant ENR with a unique YX_7_K type catalytic domain, unlike the TCL sensitive FabI and mildly TCL-resistant FabL, which contain a YX_6_K type catalytic domain and are strictly conserved only in bacteria. This study is the first to report TCL resistance in an YX_7_K type FabI2 ENR which functionally complemented *fabI* mutation and identifies FabI2 as the only known ENR shared by both prokaryotes and eukaryotes. In addition, the presence of these TCL-resistant FabI2 ENR homologs among obligate intracellular human pathogenic bacteria and obligate intracellular pathogenic Apicomplexa suggest that those organisms may remain unaffected by TCL treatment.

## Supplementary information

Supplementary information

Suppementary data
