# Supplementary material for: Biochemical and Structural Insights Concerning Triclosan Resistance in a Novel YX7K Type Enoyl-Acyl Carrier Protein Reductase from Soil Metagenome
Source: Sci Rep. 2019 Oct 28;9:15401. doi: 10.1038/s41598-019-51895-2 (PMC6817880; doi:10.1038/s41598-019-51895-2)
Supplement: Supplementary file 1 — Supplementary information [file 41598_2019_51895_MOESM1_ESM.pdf]

# Biochemical and Structural Insights Concerning Triclosan Resistance in a Novel YX$_7$K Type Enoyl-Acyl Carrier Protein Reductase from Soil Metagenome

Running title: Triclosan resistant enoyl-ACP reductase in eukaryotes and prokaryotes

Raees Khan[a,b]†, Amir Zeb[c]†, Kihyuck Choi[a], Gihwan Lee[c], Keun Woo Lee[c*], Seon-Woo Lee[a*]

[a]*Department of Applied Bioscience, Dong-A University, Busan 49315, Republic of Korea*

[b]*Department of Biological Sciences, National University of Medical Sciences, Rawalpindi 46000, Pakistan*

[c]*Division of Applied Life Science (BK21 Plus Program), Plant Molecular Biology and Biotechnology Research Center (PMBBRC), Research Institute of Natural Science (RINS), Gyeongsang National University, Jinju 52828, Republic of Korea*

*Corresponding author Seon-Woo Lee

Email: seonlee@dau.ac.kr

TEL: +82-51-200-7551

FAX: +82-51-200-7505

or

Keun Woo Lee

Email: kwlee@gnu.ac.kr

TEL: +82-55-772-1360

FAX: +82-55-772-1359

†These authors contributed equally to this work.

[Fig. S1, Raees Khan et al.]

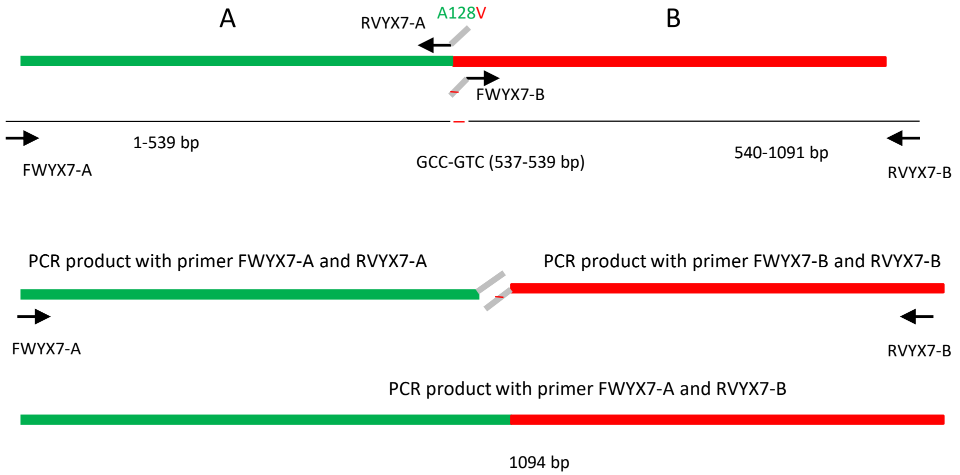


**Fig. S1. Site-directed mutagenesis using overlap extension PCR to substitute A128V.** The *FabI2* gene sequence along with additional nucleotides on the 5′ and 3′ ends (for primer designing and promoter regions) were divided into two regions; A and B. Region "A" contain the A128 encoding region of FabI2 which will be substituted with V128, already incorporated in the RVYX7-A primer. Fragments "A" and "B" were amplified using mentioned primers which carries complementary overlapping nucleotide sequences (presented as gray overhangs) for the purpose of fusion PCR. Both fragments were finally fused using primers FWYX7-A and RVYX7-B to create mFabI2 with the A128V substitution.

[Fig. S2, Raees Khan et al.]

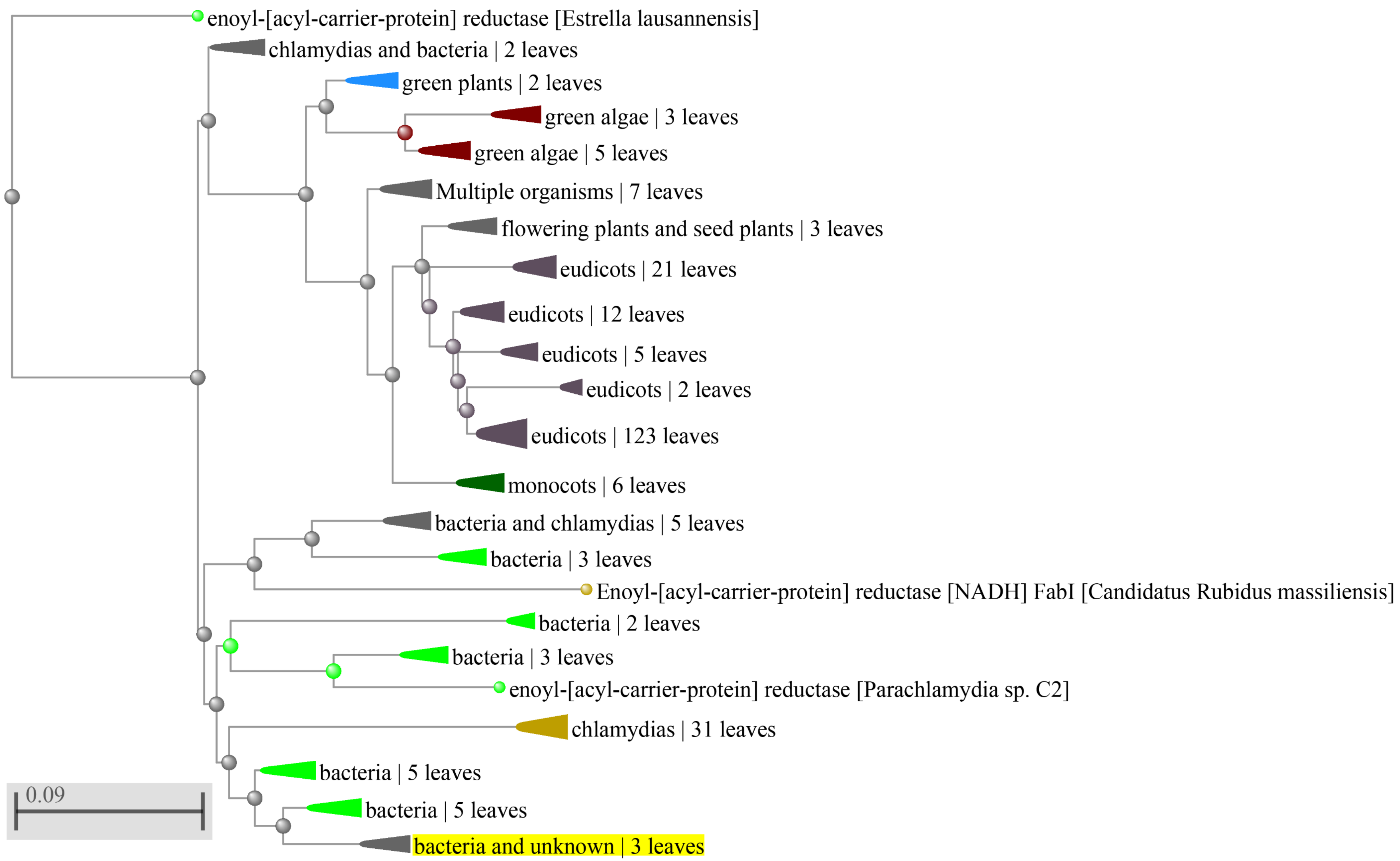


**Fig. S2. Distance tree of FabI2 ENR.** Fast minimum evolution method based phylogenetic tree was constructed using the "Distance tree of results" option of the NCBI blastp results. The closest 250 hits were selected, sharing 66-84% identity to FabI2. The maximum sequence difference was set at 0.85 and the scale bar represents 0.09 estimated amino acid substitutions per residue. Label color for the query is yellow, whereas for other closest hits various label colors has been assigned. The size of the leave represents the number of hits corresponding to it.

**[Fig. S3, Raees Khan et al.]**

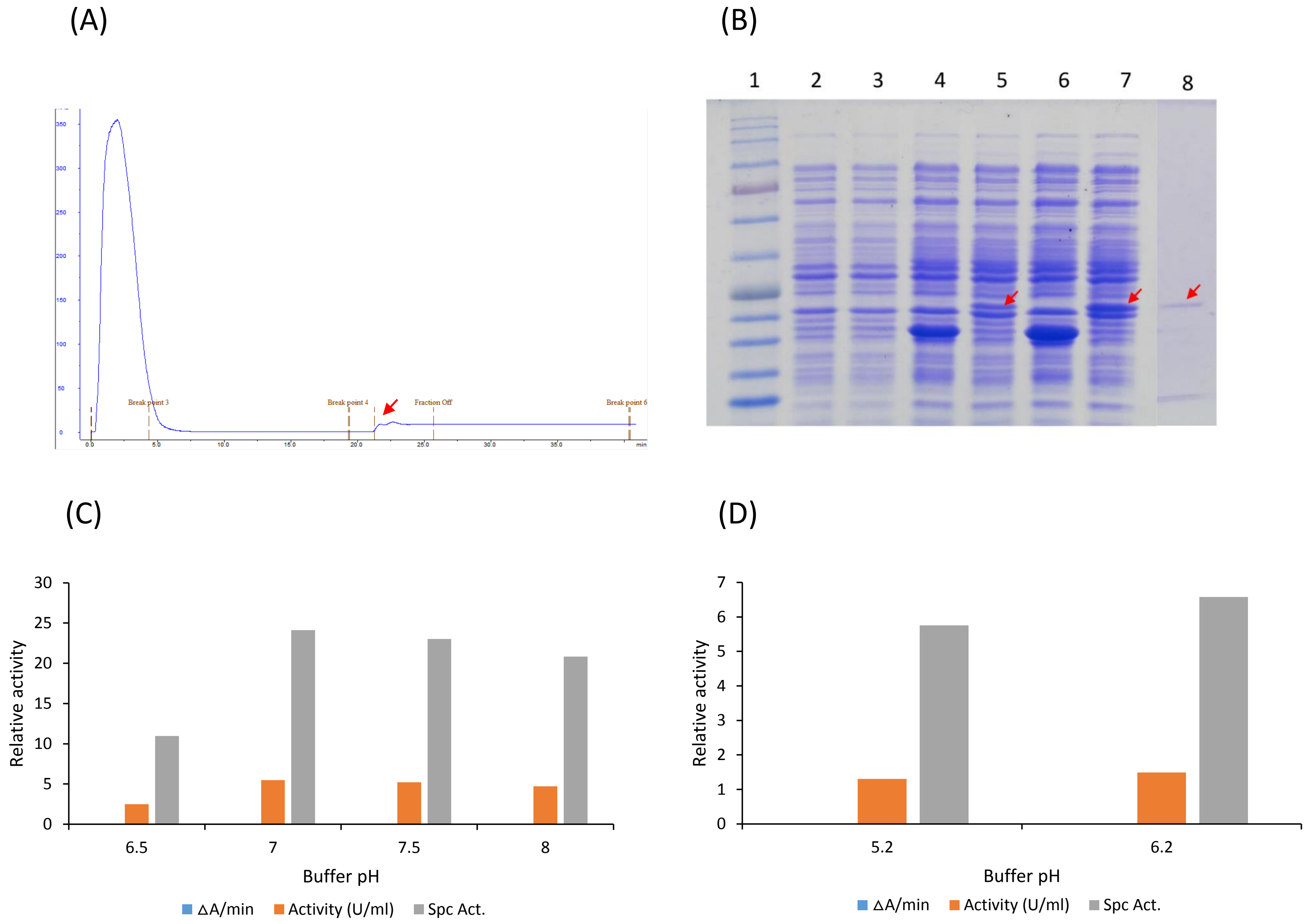


**Fig. S3. Purification of FabI2 protein.** (A) FPLC chromatogram of FabI2 protein. Red arrow indicates the chromatogram peak corresponding to desired fusion protein (B) FabI2 protein was purified by affinity chromatography. Gel electrophoresis indicate the purity of FabI2 protein. Lane 1, standards; lane 2, uninduced *E. coli* DE3 cultures (+ve control) carrying FabL2 in pET-30b(+); lane 3, Uninduced *E. coli* DE3 cultures carrying FabI2 in pET-30b(+); Lane 4, *E. coli* DE3 cultures carrying FabL2 in pET-30b(+) 2 hours post IPTG (1 mM) induction; Lane 5, *E. coli* DE3 cultures carrying FabI2 in pET-30b(+) 2 hours post IPTG (1 mM) induction; lane 6, *E. coli* DE3 cultures carrying FabL2 in pET-30b(+) 4 hours post IPTG (1 mM) induction; Lane 7, *E. coli* DE3 cultures carrying FabI2 in pET-30b(+) 4 hours post IPTG (1 mM) induction; Lane 8 purified FabI2 (37.74 kDa) protein (cropped from a separate SDS gel picture and merged here). (C) Enzyme activity of the purified FabI2 protein in 100 mM sodium phosphate buffer (pH; 6.5-8). (D) Enzyme activity of the purified FabI2 protein in 100 mM sodium citrate buffer (pH; 3.2-6.2). Highest activity was observed at pH 7.0 in case of sodium phosphate buffer and at pH 6.2 for sodium citrate buffer. Orange bar represents enzyme unit (Unit/ml) of FabI2 and gray bar represents specific activity, whereas the blue bars represents change in absorbance per minute.

**[Fig. S4, Raees Khan et al.]**

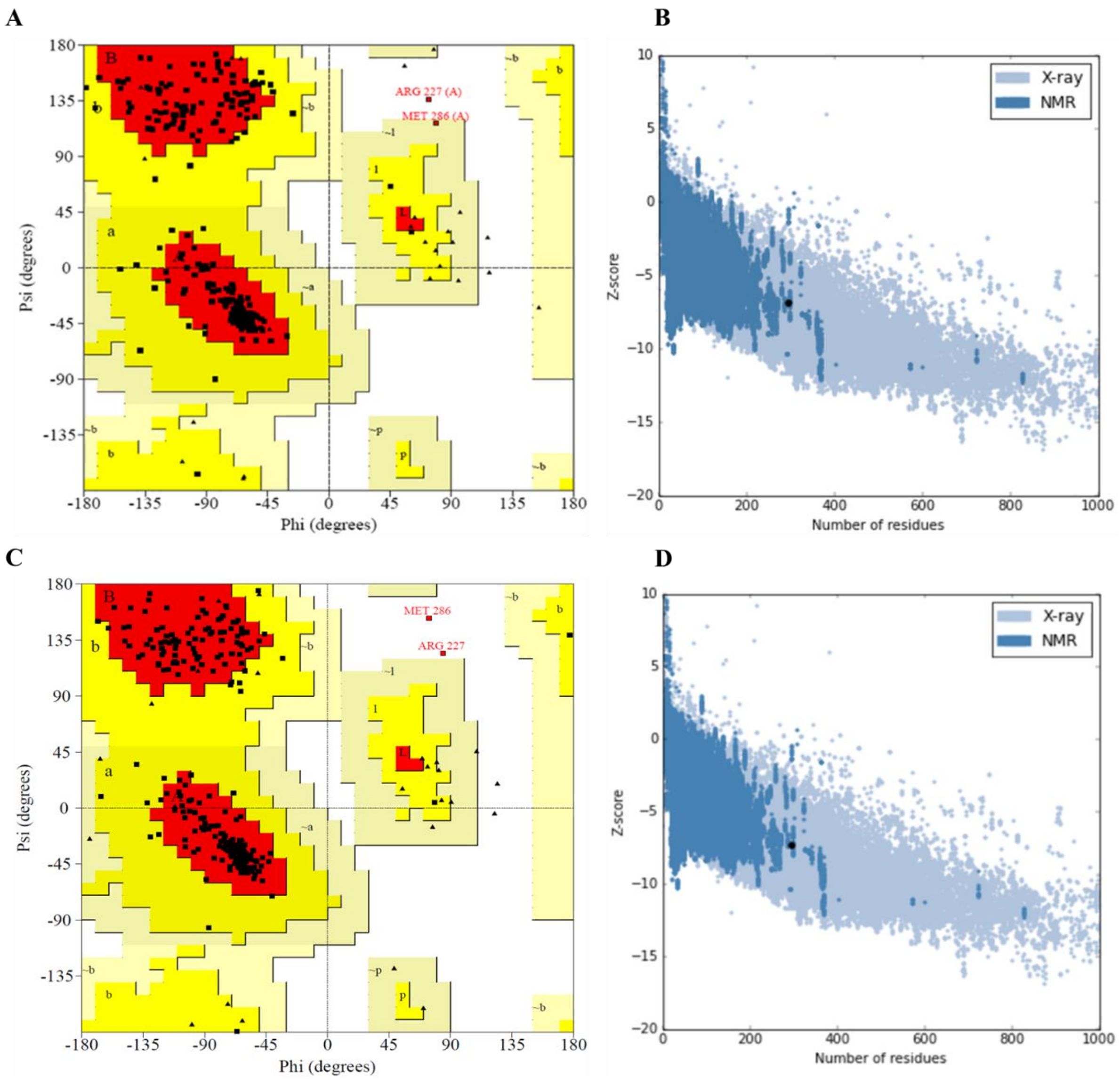


**Fig. S4. Validation of FabI2 and mFabI2 models.** Ramachandran plots of phi/psi (Φ/Ψ) distribution of homology models, calculated by PROCHECK, of (**A**) FabI2 and (**C**) mFabI2. The Z-score plot for modeled structures of (**B**) FabI2 and (**D**) mFabI2. Both the analyses suggested that modeled structures of FabI2 and mFabI2 were reasonably accurate and were within the range typically observed for native proteins of similar size.

**[Fig. S5, Raees Khan et al.]**

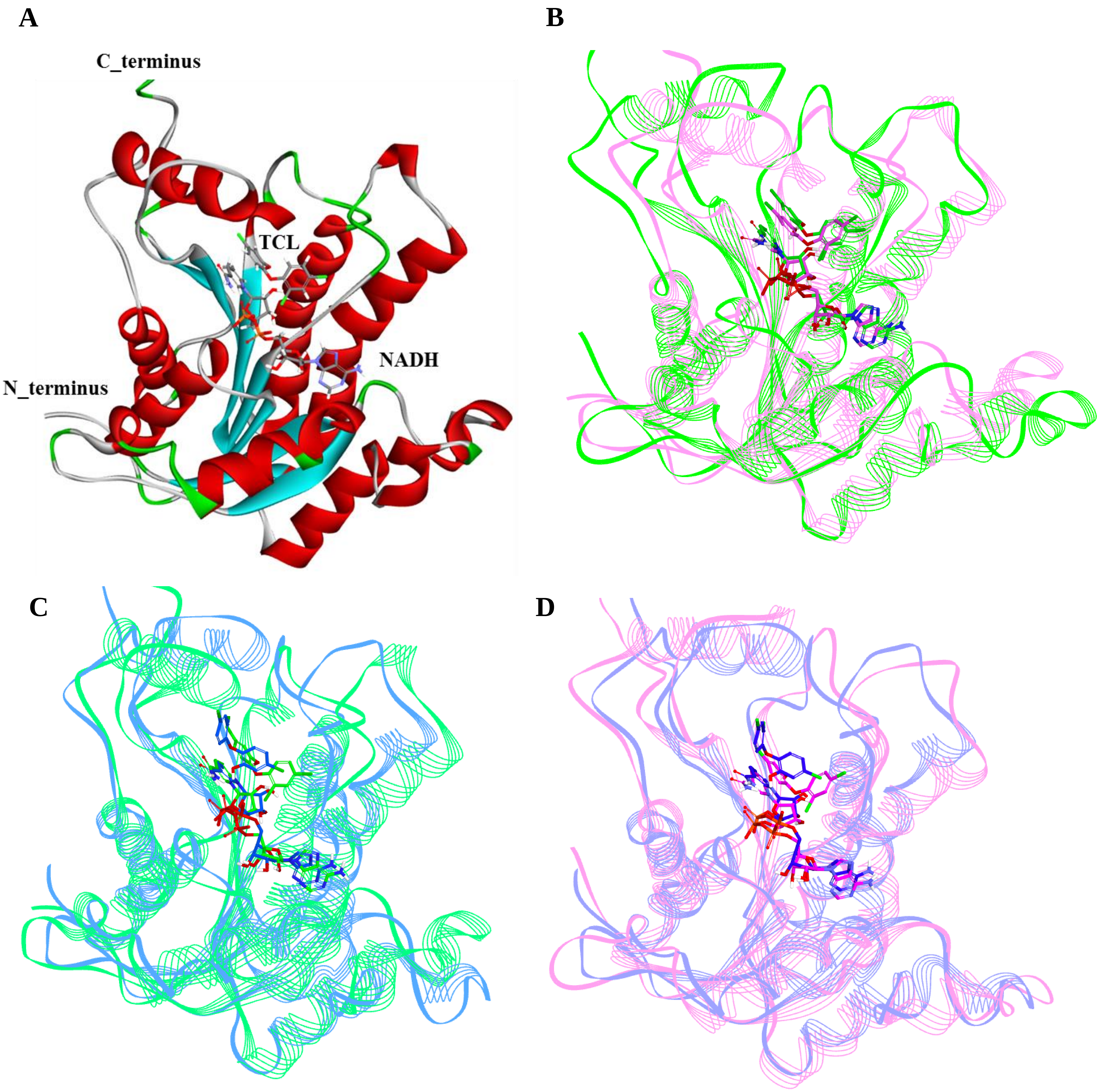


**Fig. S5. Structure of BnENR (template) and superimposition of BnENR, FabI2 and mFabI2.** (**A**) Crystal structure of *Brassica napus* Enoyl-acyl carrier protein reductase (BnENR, PDB ID: 1D7O) has been used as template for homology modeling of FabI2. NADH and TCL are depicted as sticks representations. (**B**) Superimposition of BnENR and FabI2 in complex with NADH and TCL. Structure of BnENR is shown as green wire representation, while FabI2 is depicted as magenta wire representation. Color scheme for NADH and TCL is tailing their respective protein partner and are shown as sticks representation. (**C**) Superimposition of BnENR and mFabI2 in complex with NADH and TCL. Structure of BnENR and its associated NADH and TCL is shown as green wire and sticks model, respectively. mFabI2 and its associated NADH and TCL are colored blue. (**D**) Superimposition of FabI2 and mFabI2 in complex with NADH and TCL. FabI2 and mFabI2 are depicted as wire representations, while NADH and TCL are shown as sticks. Magenta and blue colors reflect FabI2 and mFabI2, respectively.

# [Fig. S6, Raees Khan et al.]

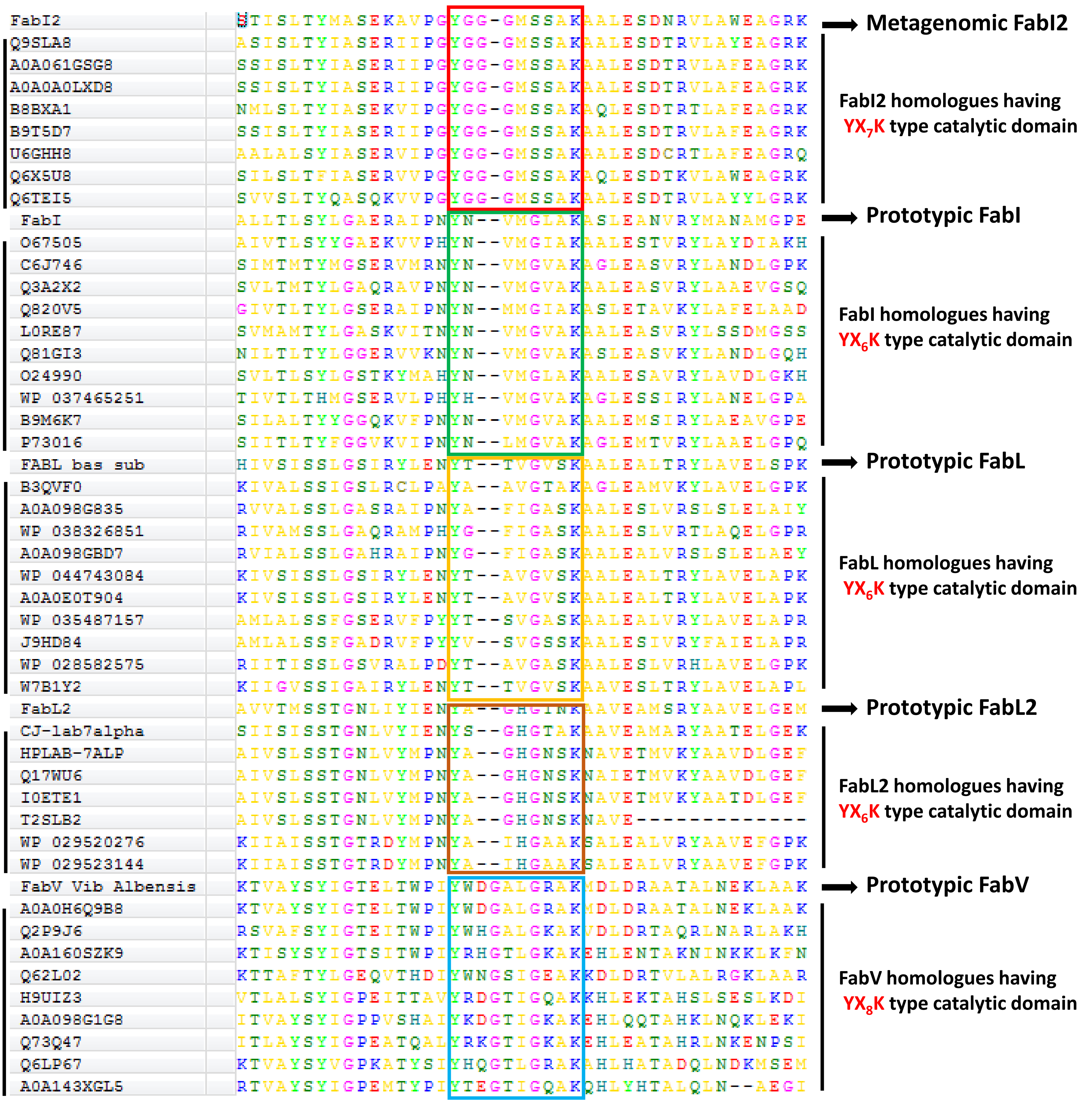


**Fig. S6. The $YX_7K$ catalytic domain was unique to metagenomic FabI2 ENR and its homologs from UniRef50 database.** Multiple alignment (partial) of metagenomic FabI2 ENR and its homologs with prototypic well characterized ENRs and their homologs from UniRef50 database. The $YX_7K$ catalytic domain (boxed in red) was unique to FabI2 ENR and was strictly conserved among its homologs. On the other hand, other prototypic ENRs has different catalytic domains; FabI ($YX_6K$ type, boxed in green ), FabL ($YX_6K$ type, boxed in dark yellow ), FabL2 ($YX_6K$ type, boxed in dark brown ), and FabV, ($YX_8K$ type, boxed in cyan).

**[Fig. S7, Raees Khan et al.]**

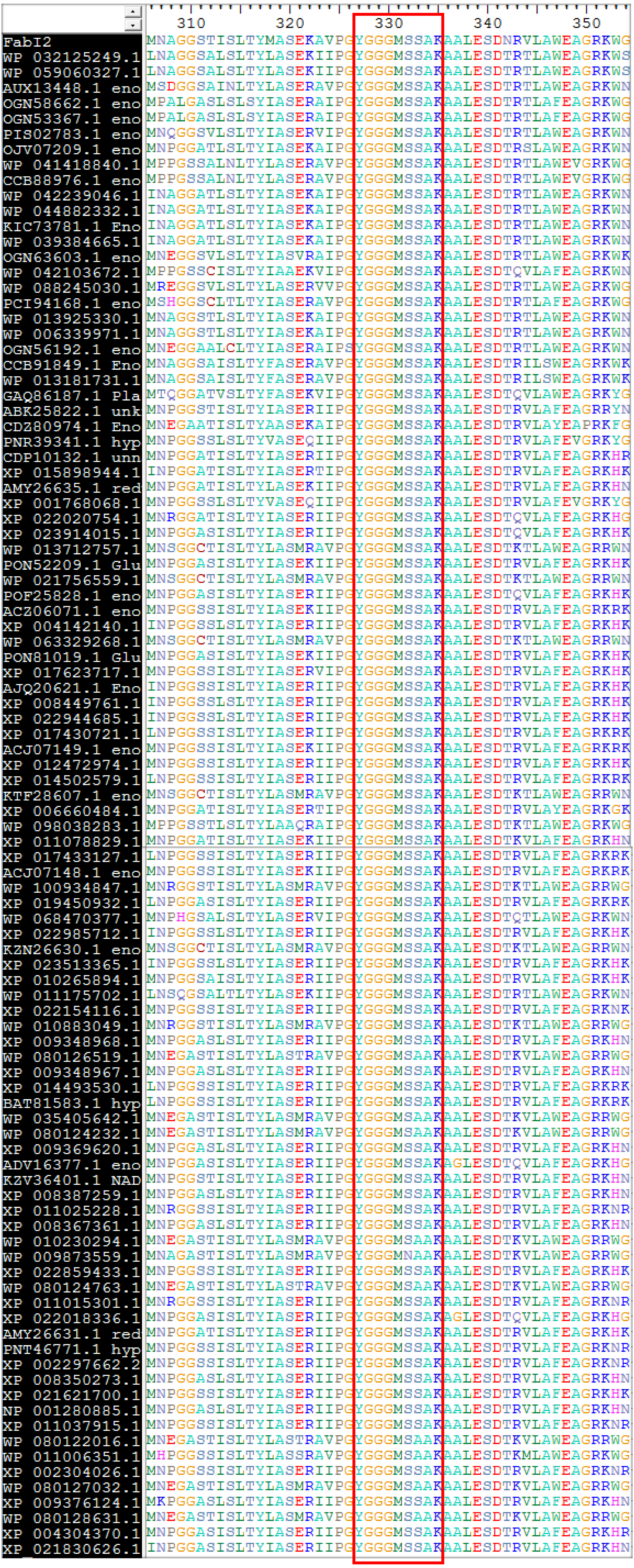

**Fig. S7. The $YX_7K$ catalytic domain was unique to metagenomic FabI2 ENR and its top 100 homologs from NCBI database.** Multiple alignment (partial) of metagenomic FabI2 ENR and its homologs (*see supplementary data 1 for the details*) with best hits from NCBI database. The $YX_7K$ catalytic domain (boxed in red) was unique to FabI2 ENR and was strictly conserved among its homologs.

# [Fig. S8, Raees Khan et al.]

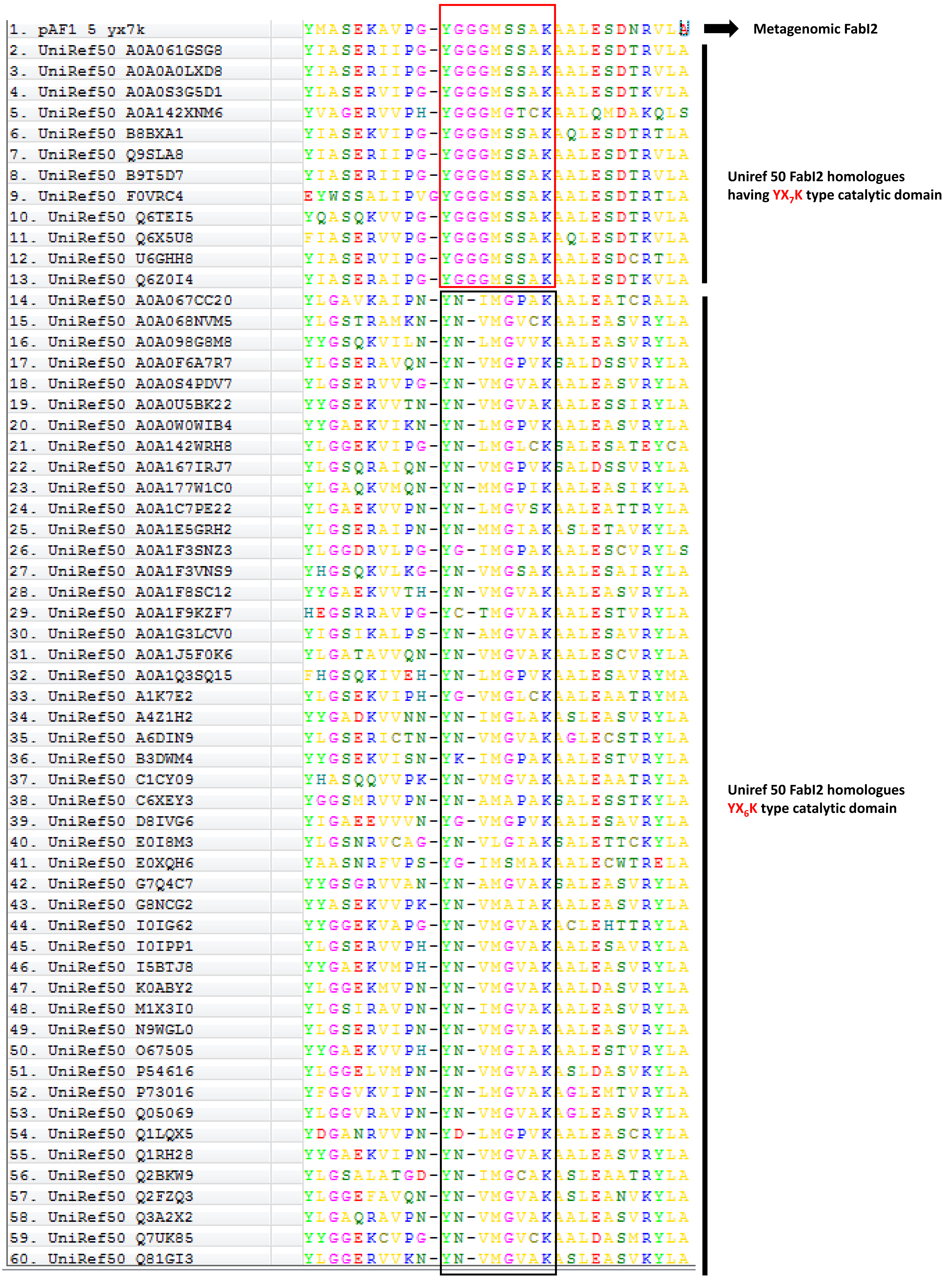


**Fig. S8. The $YX_7K$ catalytic domain was unique to metagenomic FabI2 ENR and its closely related homologs from UniRef50 database.** Multiple alignment (partially shown) of metagenomic FabI2 ENR and its homologs with best hits from UniRef50 database. The $YX_7K$ catalytic domain (boxed in red) was unique to FabI2 ENR and was strictly conserved among few of its homologs. On the other hand, most of the closely related homologues of FabI2 from UniRef50 database shared $YX_6K$ type catalytic domain (boxed in black).

**[Fig. S9, Raees Khan et al.]**

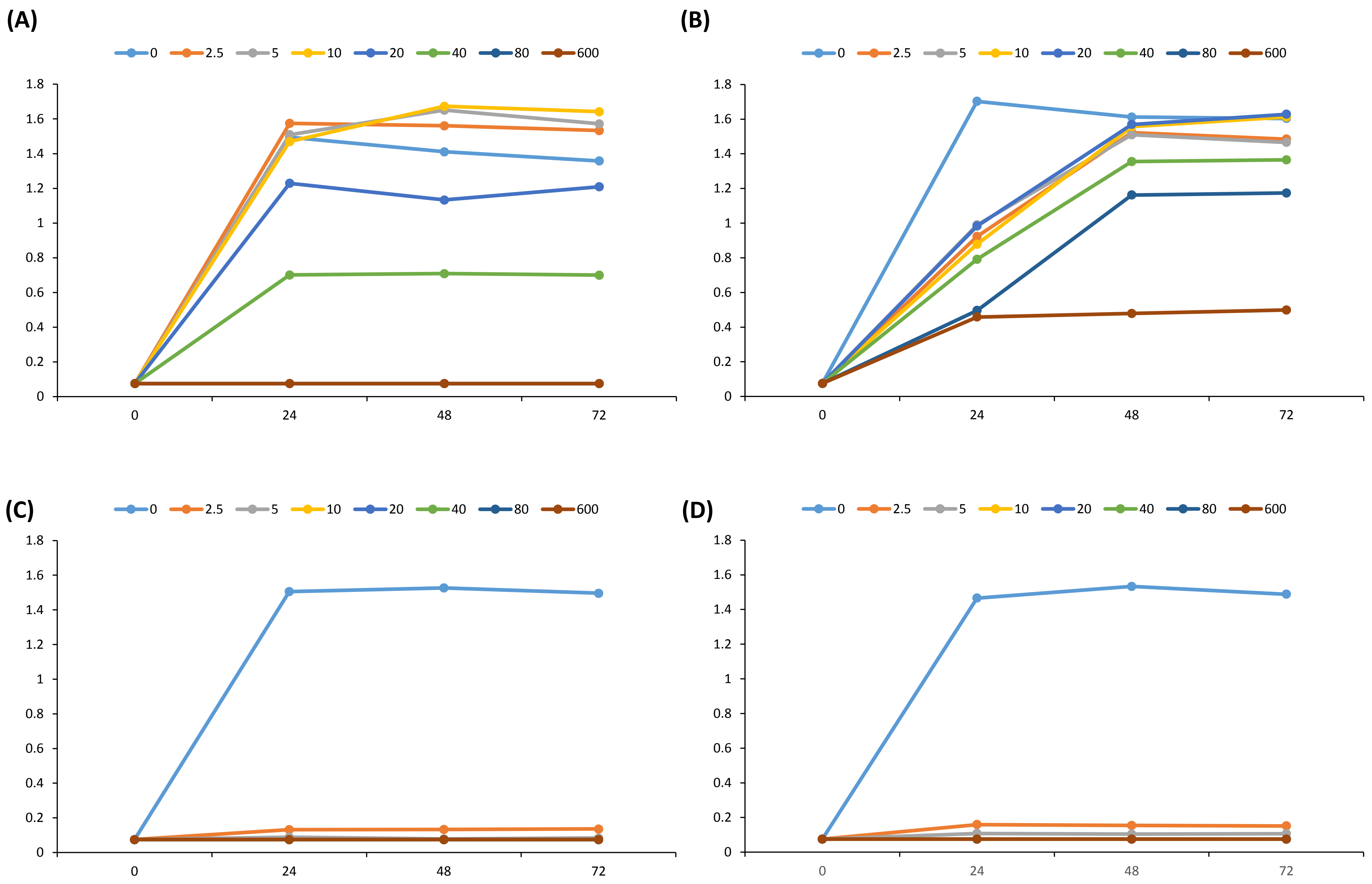


**Fig. S9. Comparative growth assay for FabI2 and mFabI2 under various concentrations of TCL.** All strains were grown in LB broth supplemented with various TCL concentrations (0–600 µg/ml). (A) Growth assay of *Escherichia coli* carrying FabI2 in pUC119. (B) Growth assay of *E. coli* carrying mFabI2 in pUC119. Two negative controls included were; (C) *E. coli* carrying prototypic FabI in pUC119 from *E. coli* K12 MG1655, and (D) *E. coli* carrying pUC119 vector only. Both of the negative controls were unable to grow at higher TCS concentrations (2.5–600 µg/ml). Legends are overlapped for higher TCL concentrations indicating no growth.

**Table S1.** Comparative identity of the metagenome derived novel FabI2 ENRs to known prototypic ENRs.

| Identity of FabI2 to known ENRs | | | | | | |
|---|---|---|---|---|---|---|
| ***Ec*FabI** | ***Vc*FabV** | ***Bs*FabL** | **FabL2** | ***S.pne*FabK** | ***Ct*FabI** | ***Tg*FabI** |
| 30% | Partial | 27% | 24% | Partial | 67% | 64 % |

**Symbols and abbreviations**; Partial, these ENRs showed very low levels of similarity, only to a small region of the target prototypic ENR; *Ec*FabI, FabI ENR from *Escherichia. coli*; *Vc*FabV, FabV ENR from *Vibrio Cholera*; *Bs*FabL, FabL from *Bacillus Subtillis;* FabL2, previously identified novel FabL like metagenomic ENR, *S.pne*FabK, FabK from *Streptococcus pneumonia*; *Ct*FabI, FabI from *Chlamydia trachomatis*; *Tg*FabI, FabI from *Toxoplasma gondii*.

**Table S2.** Kinetics of the metagenomic FabI2 ENR.

| **Substrate/cofacotr** | **Km (μM)** | ***Kcat*** **($min^{-1}$)** | ***Kcat*****/Km ($μM^{-1}min^{-1}$)** | **Vmax (μM/min)** |
|---|---|---|---|---|
| Crotonyl-CoA | 13.85 | 0.19396 | 0.014 | 4.441 |
| NADH | 18.21 | 0.9546 | 0.052 | 14.67 |

**Table S3:** Comparison of the type of $YX_nK$ catalytic domain of various enoyl-ACP reductases.

| **ENR** | **Catalytic domain type** | **Reference** |
|---|---|---|
| Most SDR members | $YX_3K$ | [1, 2] |
| FabI (*Eschericia coli*) | $YX_6K$ | [3] |
| FabL (*Bacills subtilis*) | $YX_6K$ | [4] |
| FabL2 (Metagenome/ Epsilonprtoeobacteria) | $YX_6K$ | [5] |
| FabV (*Vibrio cholerae*) | $YX_8K$ | [6] |
| FabK (*Streptococcus pneumoniae*) | FMN type | [7] |
| *Brassica napus* ENR<br>*Arabidopsis thaliana* ENR<br>*Nicotinia tabacum* ENR | $YX_7K$ | [3] |
| **FabI2** | $YX_7K$ | [This study] |

Symbols and abbreviations; X in $YX_nK$ is any amino acid

**Table S4:** MIC of TCL for wild type FabI2 and its mutant mFabI2 (A128V).

| Clone | TCL concentration in LB agar growth media (µg/ml) | | | | | | |
|---|---|---|---|---|---|---|---|
| | 5 | 20 | 50 | 100 | 200 | 400 | 600 |
| mFabI2 | + | + | + | + | + | + | + |
| FabI2 | + | + | - | - | - | - | - |

Symbols and Abbreviations: +; Positive growth, -; No growth

**References**


1. Jornvall, H. *et al*. Short-chain dehydrogenases/reductases (SDR). *Biochemistry*. **34**, 6003–6013 (1995).
2. Filling, C. *et al*. Critical residues for structure and catalysis in shortchain dehydrogenases/reductases. *J. Biol. Chem*. **277**, 25677–25684 (2002).
3. Rozwarski, D. A., Vilcheze, C., Sugantino, M., Bittman, R. & Sacchettini, J. C. Crystal structure of the Mycobacterium tuberculosis enoyl-ACP reductase, InhA, in complex with NAD+ and a C16 fatty acyl substrate. *J. Biol. Chem*. **274**, 15582–15589 (1999).
4. Heath, R. J., Su, N., Murphy, C. K. & Rock, C. O. The enoyl-[acyl-carrier-protein] reductases FabI and FabL from *Bacillus subtilis*. *J. Biol. Chem*. **275**, 40128–40133 (2000).
5. Khan, R., Zeb, A., Roy, N., Magar, R. T., Kim, H. J., Lee, K. W. & Lee, S-W. Biochemical and structural basis of triclosan resistance in a novel enoyl-acyl carrier protein reductase. *Antimicrob. Agents Chemother*. **62**, e00648–18 (2018).
6. Massengo-Tiasse, R. P. & Cronan, J. E. Vibrio cholerae FabV defines a new class of enoyl-acyl carrier protein reductase. *J. Biol. Chem*. **283**, 1308–1316 (2008).
7. Massengo-Tiassé, R. P., & Cronan, J. E. Diversity in enoyl-acyl carrier protein reductases. Cell. *Mol. Life Sci*. **66**, 1507–1517 (2009).
